# Nanoparticle‐Mediated Targeted Protein Degradation: An Emerging Therapeutics Technology

**DOI:** 10.1002/anie.202503958

**Published:** 2025-05-09

**Authors:** Andrew G. Baker, Adrian Pui Ting Ho, Laura S. Itzhaki, Ljiljana Fruk

**Affiliations:** ^1^ Department of Pharmacology University of Cambridge Tennis Court Road Cambridge CB2 1PD UK; ^2^ Department of Chemical Engineering and Biotechnology University of Cambridge Philippa Fawcett Drive Cambridge CB3 0AS UK

**Keywords:** Lysosome‐targeting chimera, Nanomedicine, PROTAC, Proteolysis‐targeting chimera, Targeted protein degradation

## Abstract

Targeted protein degradation (TPD) has emerged as a transformative therapeutic strategy for eliminating disease‐associated proteins, with relevance across disorders ranging from cancer to neurodegeneration. Since its inception nearly two decades ago, TPD has attracted strong academic and commercial interest, with multiple candidates advancing into clinical trials. Despite this progress, the field faces persistent challenges, including limited solubility, poor cellular uptake, and unpredictable structure–activity relationship of small‐molecule degraders, which complicate rational design. To address these limitations, alternative platforms such as nanoparticle‐mediated protein degraders (NanoPDs) have gained attention. First reported 17 years ago, NanoPDs harness a diverse array of materials, degradation mechanisms, and linker chemistries to achieve protein clearance through novel pathways. Although promising, their clinical translation remains constrained by barriers such as lysosomal entrapment, protein corona formation, and biocompatibility concerns. In this review, we present a comprehensive overview of the current landscape of nanoparticle‐mediated TPD. We emphasize the design principles underlying nano–bio interfaces and explore the role of proximity‐induced biology as a mechanism for orchestrating protein interactions. Finally, we highlight critical challenges and key questions that must be addressed to fully realize the therapeutic potential of NanoPDs.

## Graphical abstract

Targeted protein degradation (TPD) has emerged as a powerful therapeutic approach, with numerous candidates molecules now advancing into clinical development. Recent innovations have incorporated nanoparticles to facilitate and enhance these degradation processes, yielding synergistic effects. This work provides a comprehensive overview of the current landscape of nanoparticle‐mediated targeted degradation, outlining recent advances, key challenges, and future opportunities to guide progress in this rapidly evolving field.

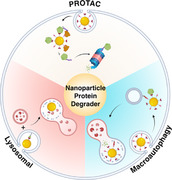

## 1 Targeted Protein Degradation

Targeted protein degradation (TPD) has emerged as a promising strategy to target dysfunctional proteins that are undruggable with current occupancy‐based inhibitors.^[^ ^1^, ^2^, ^3^, ^4^ ^]^ In this approach, a molecule is designed to bring a target protein into proximity with a component of the cell's degradation machinery to drive target degradation. This strategy has numerous benefits over conventional inhibitors, which need to occupy a functional site on the target in a stoichiometric manner to disrupt activity, in contrast, degraders can in theory bind to any site on the protein's surface. Moreover, by eliminating the target protein, these molecules block all functions, and degradation results in a prolonged effect, recovery from which requires new protein synthesis.^[^ ^5^, ^6^ ^]^ Additionally, degraders can act on multiple target molecules and therefore work substoichiometrically (referred to as event‐driven pharmacology), enabling the use of lower therapeutic doses and consequently reducing the risk of off‐target effects.^[^ ^5^ ^]^

### 1.1 PROteolysis‐TArgeting Chimeras (PROTACs)

An example of TPD molecules are PROteolysis‐TArgeting Chimeras (PROTACs), bifunctional molecules that binds simultaneously to a protein of interest (POI) and an E3 ubiquitin ligase (referred to subsequently as E3), driving target ubiquitination and subsequent degradation by the proteasome (the ubiquitin–proteasome system (UPS)).^[^ ^6^, ^7^ ^]^ The first PROTAC, Protac‐1, was developed in 2001 and was composed of a small molecule ligand of the target methionine aminopeptidase‐2 (MetAP‐2) and a peptide ligand of the E3 Skp1‐Cullin‐F box complex SCF^β‐TRCP^.^[^ ^7^ ^]^ It was not until 2008 that the first small molecule PROTAC was synthesized.^[^ ^8^ ^]^ It consisted of a selective androgen receptor modulator (SARM) and a ligand (nutlin) for the E3 MDM2 connected by a polyethylene glycol (PEG) linker. Since this report, many PROTACs have been synthesized, some of which such as Arvinas’ androgen receptor degrader bavdegalutamide (ARV‐110) have reached clinical trials, whereas others such as vepdegestrant (ARV‐471) (Arvinas and Pfizer) targeting the estrogen receptor have received FDA fast‐track designation. Furthermore, PROTACs are being further modified and enhanced with elements that are responsive to X‐rays or hypoxia to enable their selective activation at precise times.^[^ ^9^ ^]^ Despite such successes, these molecules still face challenges such as rapid elimination, low solubility, and poor cellular permeability.^[^ ^5^, ^6^, ^10^, ^11^, ^12^ ^]^ Also, despite the attempts that have been made to expand the E3 repertoire and the range of targets, there are still many E3s and targets without small‐molecule ligands.^[^ ^11^, ^13^, ^14^ ^]^

### 1.2 Molecular Glues

Molecular glues have emerged as an exciting alternative to PROTACs in TPD strategies.^[^ ^15^ ^]^ Unlike PROTACs, molecular glues are smaller and hence, more cell permeable as they are single molecule that glue two protein surfaces, typically the target and E3 ligase, together. However, their development remains challenging as most molecular glues have been discovered serendipitously rather than through rational design.^[^ ^15^ ^]^ Notable examples include the immunomodulatory imide drugs (Imidis), such as thalidomide, lenalidomide, and pomalidomide, all of which bind to the E3 ligase cereblon (CRBN).^[^ ^6^, ^16^ ^]^ This class of drugs has had significant clinical impact and is currently used in the treatment of multiple myeloma.^[^ ^17^ ^]^

### 1.3 Lysosome‐Targeting Chimeras (LYTACs)

As PROTACs only degrade intracellular targets, the Bertozzi group in 2020 introduced a strategy for the targeted degradation of extracellular (membrane‐bound or secreted) proteins using a bifunctional molecule called lysosome‐targeting chimeras (LYTACs).^[^ ^18^ ^]^ Here, an antibody for a POI is functionalized with a chemically synthesized glycopeptide ligand agonist for the cation‐independent mannose‐6‐phosphate receptor (CI‐M6PR), a common cell surface receptor. Simultaneous binding to POI and CI‐M6PR receptor results in internalization of the POI and its degradation through the endolysosomal system.^[^ ^18^ ^]^ This strategy was expanded to harness the asialoglycoprotein receptor (ASGPR), a liver specific targeting receptor to degrade specific membrane and secreted proteins in the liver.^[^ ^19^, ^20^, ^21^ ^]^ MoDE‐As exploit a similar mechanism; however, as opposed of antibody conjugates, they are small molecules.^[^ ^21^ ^]^ Bifunctional molecules harnessing other cell surface receptors have also been used for membrane and secreted (extracellular) targeted protein degradation (eTPD). These strategies include KineTACs that utilize cytokine receptors, TransTACs that harness transferrin (responsible for iron transport within the body), and EndoTags that use de novo designed miniproteins to harness ASGPR, insulin‐like growth factor 2 receptor (IGF2R), sortilin, and transferrin receptors.^[^ ^22^, ^23^, ^24^ ^]^ For a more extensive review of extracellular targeted protein degradation (eTPD) see Refs. [5, 25].

### 1.4 Sweeping Antibodies

In an alternative eTPD strategy, “sweeping antibodies”, as reported by Igawa et al., have been used to eliminate secreted POIs, such as the soluble form of interleukin 6 receptor (IL‐6R).^[^ ^26^ ^]^ These antibodies bind to the POI and pull it into the endolysosomal system for degradation. They are enhanced relative to the parent antibodies by incorporating a pH‐switchable region, which enable antibody recycling from the lysosome back to the membrane, allowing them to degrade further POI molecules. This innovative strategy proved highly successful and recently received FDA approval for the treatment of neuromyelitis optica spectrum disorder, a relapsing inflammatory disease of the central nervous system.^[^ ^27^ ^]^

### 1.5 Antibody‐Based Proteolysis‐Targeting Chimeras (AbTACs)

Antibody‐based proteolysis‐targeting chimeras (AbTACs), proteolysis‐targeting antibodies (PROTABs) that exploit E3s, and receptor elimination by E3 recruitment (REULR) use combinations of single‐domain antibodies (nanobodies) or bispecific antibodies whereby one arm binds to the extracellular domain of a transmembrane E3 (such as RNF43 and ZNRF3) and the other arm binds to the extracellular domain of a transmembrane POI.^[^ ^28^, ^29^, ^30^ ^]^ This brings the E3 and POI into proximity, leading to ubiquitination of the intracellular domain of the POI, which directs it for lysosomal or proteasomal degradation.^[^ ^5^, ^28^, ^31^ ^]^

### 1.6 Harnessing the Autophagy Pathway

The targeted protein degradation field has expanded to encompass the autophagy‐lysosome pathway, for example, autophagy‐targeting chimers (AUTACs),^[^ ^32^ ^]^ AUTOphagy‐TArgeting Chimera (AUTOTACs),^[^ ^33^ ^]^ and autophagy‐tethering compounds (ATTECs).^[^ ^34^ ^]^ This degradation strategy has been described as macroautophagy degradation targeting chimeras or MADTACs.^[^ ^35^ ^]^ Because they exploit autophagy, these molecules have the potential to target both intracellular proteins and bigger macromolecules such as protein aggregates and organelles.^[^ ^35^ ^]^

### 1.7 Nanoparticle‐Mediated Targeted Protein Degraders (NanoPDs)

At around the same time as the first small molecule PROTACs, in 2008, nanoparticles emerged as tools for extracellular protein degradation with the development of gold nanoparticles decorated with anti‐Her2 antibodies that induced the degradation of the HER2 receptor.^[^ ^36^ ^]^ This nanoparticle system facilitated the internalization and subsequent degradation of HER2 receptor in a human breast cancer cell line (SK‐BR‐3).^[^ ^36^ ^]^ HER2 degradation was found to be dependent on the nanoparticle size, with 40–60 nm particles demonstrating the highest efficiency.

Since this initial work, modulation of membrane‐bound and intracellular proteins has been demonstrated with nanoparticles of different sizes functionalized with RNA aptamers, peptides, antibodies, and small molecules.^[^ ^37^, ^38^, ^39^ ^]^ In the majority of these studies, degradation occurs through lysosomal‐degradation pathways as nanoparticles are readily taken up into lysosomes. Recently, likely in response to the growing interest in targeted protein degradation technologies, nanoparticles have been utilized as nanocarriers of protein binders and mediators of the protein degradation.^[^ ^40^ ^]^ A variety of materials have been explored to date, including DNA nanostructures, gold clusters, and liposomal nanoparticles.^[^ ^38^, ^41^, ^42^ ^]^ Despite these advancements, challenges in designing effective and functional bio–nano interfaces remain a significant barrier to clinical translation.

Compared to small molecules and biologics, nanoparticles face greater challenges in gaining clinical approval.^[^ ^43^, ^44^ ^]^ However, there are specific instances where nanoparticles offer distinct advantages over small molecules. For example, they enable prolonged circulation times, which can enhance therapeutic effects such as is the case with Doxil, a liposomal formulation of doxorubicin.^[^ ^45^ ^]^ They often may also have innate physical properties for therapy such as NBTXR3/Hensify, a hafnium oxide‐based nanoparticle that enhances radiotherapy.^[^ ^46^ ^]^ In addition, there is the potential to functionalize nanoparticles with multiple molecules simultaneously enabling multimodal imaging and targeted delivery. For example, Cornell Dots, used in phase I and II clinical trials, are a hybrid mesoporous silica nanoparticles that were functionalized with both a targeting ligand and a radiolabel for PET imaging.^[^ ^44^, ^47^ ^]^

As a result, nanoparticle‐mediated degraders may present significant benefits over small‐molecule or biologics‐based degraders (Table 1). Unlike traditional small molecule or biologic approaches, nanoparticles feature different sizes and possess multiple binding sites meaning that a single nanoparticle can bind to a large number of proteins simultaneously. Additionally, various nanomaterials can be leveraged to enhance protein degradation. Further, studies could also utilize innate properties of nanomaterials, such as those for imaging.

**Table 1:** Summary of TPD strategies.

Degradation technique	Target class	Design considerations	Extent of translation
PROTACs	Intracellular proteins	Synthesizable, but high MW	Phase III
Molecular glues	Intracellular proteins	Hard to synthesize; most discovered serendipitously	FDA approved
LYTACs	Extracellular (membrane‐bound and soluble proteins	Antibody conjugates	Preclinical
AbTACs	Membrane proteins	Antibody conjugates	Preclinical
Sweeping antibodies	Soluble extracellular proteins	Antibodies	FDA approved
MADTACs	Intracellular proteins and larger biomolecules	Synthesizable, high MW	Preclinical
Nanoparticle degraders	Intracellular, extracellular, soluble, and larger biomolecules	Adaptable, nanomaterials	Preclinical

MW, molecular weight

Although the field of NanoPDs is still in its infancy and limited to preclinical studies, it presents numerous opportunities to outperform traditional small molecule or biologic therapies. This review provides an overview of the field to date, with a focus on degradation strategies, materials utilized, and attachment approaches taken. We conclude by discussing the challenges and opportunities arising from the integration of nanotechnology and proximity‐induced biology.

## 2 Nanoparticle‐Mediated Protein Degradation Strategies

**Figure 1:**
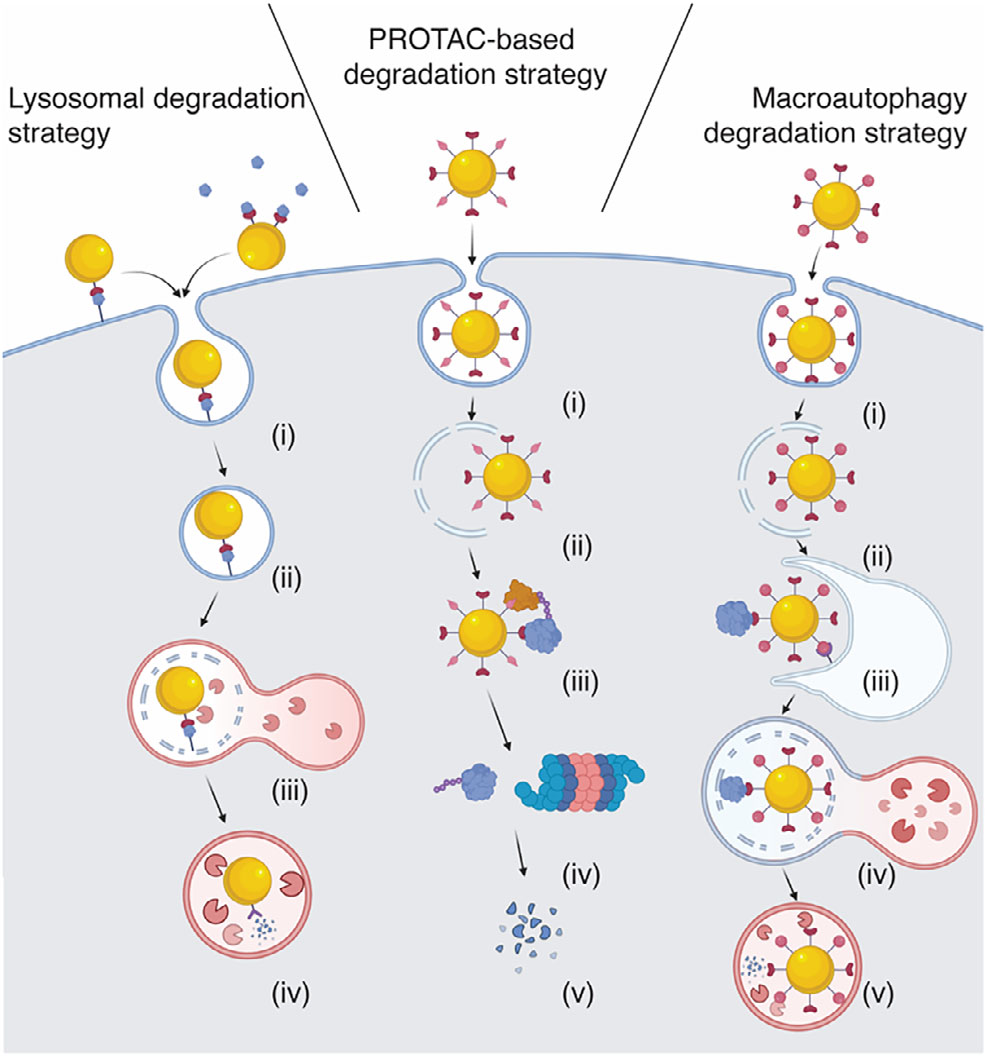
Overview of protein degradation mechanisms in nanoparticle‐mediated protein degradation strategies. a) Lysosome‐based degradation strategy whereby i) the nanoparticle binds to a transmembrane or extracellular proteins of interest (POIs), ii) complex is internalized into the cell through endocytosis, iii) resulting endosome fuses with a lysosome to form endolysosome, in which iv) POI is degraded by lysosomal enzymes. b) In PROTAC‐based NanoPD strategy, i) the nanoparticle enters the cell by endocytosis, then ii) it escapes from the endosome into the cytosol and iii) binds to a cytosolic POI and E3 ligase complex through its ligands, enabling ubiquitination of the POI. The ubiquitinated POI is then iv) transported to the proteasome where v) degradation occurs. c) Autophagy‐lysosomal‐based degradation strategies also involves i) endocytosis of nanoparticles, which must ii) escape into the cytosol iii) to bind to the cytosolic POI and autophagosome. iv) The NanoPD‐incorporated autophagosome then fuses with lysosome to form an autolysosome resulting in the v) degradation of the POI.

Initially, nanoparticle‐mediated protein degraders primarily relied on lysosomal uptake and subsequent degradation as the mode of action. However, they have since expanded to utilize the UPS, whereby ligands for the POI and for an E3 are immobilized on the surface of the nanoparticle facilitating POI degradation by the proteasome (Figure 1).

### 2.1 Lysosome Degradation Strategy

The lysosome degradation‐based strategy exploits the cell's intrinsic tendency to traffic nanoparticles into lysosomes. When nanoparticle‐mediated protein degraders (NanoPDs) bind to a transmembrane protein, the cell responds by internalizing both the nanoparticle and the bound protein through endocytosis. This is followed by the fusion of the NanoPD‐containing endosome with a lysosome, forming an endolysosome. In this environment, the nanoparticle functions as a lysosomal‐targeting agent, requiring only mono functionalization with a ligand that binds the POI (Figure 1). Despite the simplicity of this nanoparticle functionalization, the lysosomal degradation strategy is limited to targeting membrane‐bound surface proteins or soluble proteins (Table 1). However, following the first study,^[^ ^36^ ^]^ the nanoparticle degradation approach has expanded beyond just surface proteins to include extracellular soluble proteins such as cytokines and chemokines and extracellular vesicles,^[^ ^48^, ^49^ ^]^ similar to the action of “sweeping antibodies”.^[^ ^5^, ^18^, ^26^ ^]^

### 2.2 UPS Degradation Strategy (PROTAC)

Unlike lysosome‐based degradation strategies, UPS degradation strategy (PROTAC‐based systems) requires both a POI‐binding ligand and an E3‐binding ligand for degradation of intracellular targets.^[^ ^1^, ^7^ ^]^ In comparison to small molecule PROTACs, nanoparticles offer the advantage of presenting multiple copies of each ligand on the surface, thereby concentrating them and increasing valency.^[^ ^50^ ^]^ To date only two E3 ligase have been used for NanoPDs, the von Hippel–Lindau (VHL) and the cereblon (CRBN) cullin‐RING E3 ligases, which are also the most commonly used in small molecules approaches.^[^ ^6^, ^13^ ^]^ One potential limitation is that after endocytosis of the nanoparticles, endosomal escape is required, which can be difficult to achieve. The approach has been applied to a variety of intracellular protein targets such as cyclin dependent kinases (CDK4 and 6) as well as antiapoptotic proteins (BcL‐xL) and has employed diverse materials, including gold nanoparticles, DNA nanostructures, and liposomes.^[^ ^36^, ^41^, ^51^, ^52^ ^]^

### 2.3 Autophagy‐Lysosomal Degradation Strategy

Like the PROTAC method, the autophagy‐lysosomal or macroautophagy degradation (MADTAC) strategy requires two types of ligands, one for the POI and one for a component of the autophagy system. These ligands promote the accumulation of autophagosomes and facilitates the binding of the POI‐loaded nanoparticle to the autophagosome, ultimately driving POI degradation (Figure 1).^[^ ^33^, ^53^ ^]^ In contrast to other strategies, MADTAC‐based NanoPDs should be able to degrade both intracellular proteins and larger cytosolic structures. Although the precise mechanism is still under investigation, it is hypothesized that nanoparticles initially enter the endolysosomal system and subsequently exit into the cytosol. Once in the cytoplasm, the nanoparticles bind to the POI and induce autophagy, surrounding the nanoparticle and its target protein. The resulting autophagosome then fuses with a lysosome, leading to the degradation of the POI. Although this mechanism has been validated in only a few studies to date, it represents a potentially valuable strategy that warranting further exploration in future experiments.^[^ ^53^, ^54^ ^]^

## 3 Nanoparticles Used for Targeted Protein Degradation

**Figure 2:**
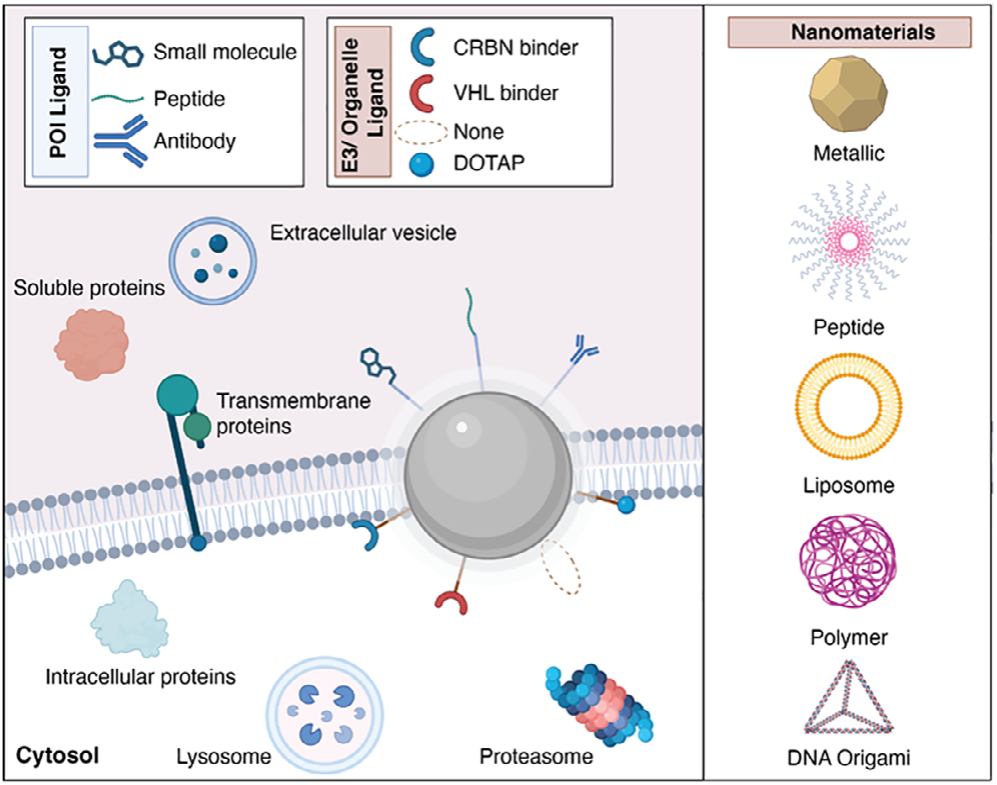
Nanostructured materials, representative ligands, and proteins of interest (POIs) used in design of nanoparticle‐mediated protein degraders. CRBN, cereblon; VHL, von‐Hippel–Lindau; DOTAP, 1,2‐dioleoyl‐3‐trimethylammonium‐propane.

First developed in 2008, nanoparticle‐mediated protein degradation has seen rapid expansion in the past two years, driven by the growing interest in the field of targeted protein degradation (Figure 2 and Table 2). These systems employ a wide variety of materials, including gold nanoparticles, polymers, liposomes, micelles, and DNA‐based nanoparticles, highlighted below, which showcase the versatility of this approach in utilizing different degradation routes.

**Table 2:** Summary of nanoparticles used in design of protein degraders.

Nanomaterial	POI ligand	Degrader ligand	Conjugation method	Target protein	Degradation pathway	Size (nm)
Gold nanoparticles^[^ ^36^ ^]^	Anti‐Her2 antibody	n/a	Adsorption	HER2	Lysosome	2–100
Gold nanostars^[^ ^38^ ^]^	Anti‐HER2 aptamer	n/a	Au‐S	HER2	Lysosome	40
Gold nanoparticles^[^ ^59^ ^]^	Ceritinib	Pomalidomide	Au‐S	ALK	PROTAC	30
Gold nanoclusters^[^ ^60^ ^]^	HER2‐binding peptide	Thalidomide	Au‐S	HER2	PROTAC	2–3
Tetrapeptide assemblies^[^ ^63^ ^]^	Tamoxifen, enzalutamide, palbociclib, JQ1, mirdametinib, dasatinib, gefitinib	VHL ligands 2, thalidomide	Peptide coupling	ERα, AR, CDK4, CDK6, BRD2, BRD4, MEK1, MEK2, BCR‐ABL, EGFR	PROTAC	50–200
Tetrapeptide assemblies^[^ ^42^ ^]^	Tamoxifen, enzalytamide, cobimetinib, dasatinib	HSC70‐binding peptide	Peptide coupling	ERα, AR, MEK1, MEK2, BCR‐ABL	Lysosome	100–300
Amphiphilic peptide assemblies^[^ ^66^ ^]^	EGFR and CD24 Antibody	Asialoglycoprotein receptor	NHS coupling	EGFR and CD24	Lysosome	200
Peptide β‐sheet^[^ ^67^ ^]^	EGFR ligand	VHL ligand 2	Alkyne–azide click reaction	EGFR, AR	PROTAC	n/a
Peptide β‐sheet^[^ ^68^ ^]^	BcL‐xL binding Peptide	VHL peptide ligand	Peptide coupling	BcL‐xL	PROTAC	40
Peptide self‐assembly^[^ ^69^ ^]^	IL‐17A binding ‐peptides	SRA‐binding peptide	Peptide coupling	IL‐17A, PD‐L1	Lysosome	150
Liposome^[^ ^51^ ^]^	4‐hydrotamoxifen	VHL ligand 2	NHS coupling	ERα	PROTAC	150
Liposome^[^ ^52^ ^]^	Mirdametinib and ceritinib analogs	VHL ligand	NHS coupling	Alk, MEK	PROTAC	150
Red blood cell membrane vesicle‐fused liposome^[^ ^71^ ^]^	CD47 on surface of membrane	n/a	Derived vesicle coextruded with liposome	SIRPα	Lysosome	120
Genetically engineered exosomes^[^ ^72^ ^]^	HER2 and PD‐L1 antibody	Asialoglycoprotein receptor	Genetically engineered exosome	HER2 and PD‐L1	Lysosome	120
Bacterial outer membrane vesicles^[^ ^73^ ^]^	PD‐L1 antibody and mannose 6‐phosphate receptor ligand	Mannose 6‐phosphate receptor	Biotin and streptavidin	PD‐L1	Lysosome	100
Polystyrene nanoparticles^[^ ^48^ ^]^	PD‐L1 antibody, MMP‐2 antibody and annexin V	n/a	Biotin and streptavidin	PD‐L1, MMP‐2, extracellular vesicles	Lysosome	100
PLGA, liposome, lipopolyplex, exosome, PEG, red blood cell membrane bound dextran, AuNP^[^ ^39^ ^]^	Nimotuzumab, cetuximab, camrelizumab, atezolizumab, pertuzumab, inetetamab, ACE2 and CD13‐binding peptide	n/a	NHS coupling	EGFR, PD‐1, PD‐L1, HER2, ACE2, CD13, CD4	Lysosome	100–200
Mal‐PEG‐PLA^[^ ^53^ ^]^	Mutant p53‐binding peptide (MBP)	DOTAP	Cysteine–maleimide (MBP)/hydrophobic self‐assembly	Mutant p53	MADTAC	100
PLGA^[^ ^75^ ^]^	Mouse PD‐L1 antibodies	n/a	NHS coupling	Murine PD‐L1	Lysosome	50
Semiconducting polymer^[^ ^76^ ^]^	Interleukin 4 receptor peptide	Lysosome sorting peptide	Peptide synthesis	Interleukin 4 receptor	Lysosome	40
Chitosan based^[^ ^54^ ^]^	Amyloid β‐ binding peptides	Autophagy inducing peptide	Cysteine linkages	amyloid β	MADTAC	30–50
DNA tetrahedron^[^ ^37^ ^]^	Anti‐HER2 aptamer	n/a	DNA self‐assembly	HER2	Lysosome	20
DNA tetrahedron^[^ ^79^ ^]^	Aptamer SL1	Cell membrane binder cholesterol	DNA self‐assembly	c‐Met	Lysosome	12
DNA tetrahedron^[^ ^41^ ^]^	Palbociclib, atuveciclib, HPK1 ligand	Pomalidomide	DBCO‐azide click reaction, maleimide‐amine coupling	CDK6, CDK9, ERG, HPK1	PROTAC	10
DNA tetrahedron^[^ ^80^ ^]^	STAT3 DNA decoy sequence	VHL ligand	DBCO‐azide click chemistry	STAT3	PROTAC	10–20
Circular DNA origami^[^ ^79^ ^]^	Anti‐EGFR and PDL1 antibodies	n/a	DNA self‐assembly after antibody modification	PD‐L1 and EGFR	Lysosome	15–30
Carbon dot^[^ ^82^ ^]^	BMS‐1166	Thalidomide	EDC‐NHS coupling	PD‐L1	PROTAC	3
Nanots^[^ ^49^ ^]^	sPD‐L1	n/a	n/a	sPD‐L1	Lysosome	n/a
Protein based	PD‐L1	Cation‐independent mannose 6‐phosphate receptor	in situ polymerization	PD‐L1	Lysosome	30

### 3.1 Gold Nanoparticles

Gold nanoparticles (Au NPs) have been used in nanomedicine both to design successful biosensing platforms and to enable clinical imaging (i.e., as CT contrast agents)^[^ ^44^, ^55^ ^]^ and photothermal ablation of cancer tissue.^[^ ^56^ ^]^ Their popularity stems from favorable properties and well‐established synthesis protocols that offer control over the size and shape, as well as the surface modification. Thiol ligands have been extensively used for modification of gold surface and many ligands are commercially available, easing the functionalization of this material.

The first use of Au NPs for TPD was reported by Jiang and coworkers in 2008, showing that hepcidin‐functionalized Au NPs guide the internalization and degradation of HER2 in a human breast cancer cell line (SK‐BR‐3) through the lysosomal pathway.^[^ ^36^ ^]^ Interestingly, the same study found that degradation is the most efficient using 40–60 nm NPs, which was most likely caused by the physical limitations of the endocytic cavities and engagement of multiple surface receptors.^[^ ^36^ ^]^ It was hypothesized that the smaller (<40 nm) NPs would not engage with multiple receptors, whereas the larger ones (>60 nm) do not interact with the receptors at the right distances. Following this first study, Lee et al. developed Au nanostars employing a HER2‐specific DNA aptamer as targeting ligand.^[^ ^38^ ^]^ More recently in 2024, Liu et al. demonstrated Au NPs modified with anti‐EGFR antibody nimotuzumab that were able to degrade EGFR through the lysosomal route.^[^ ^39^ ^]^

Despite their versatility, few researchers have investigated how tuning the physical properties of the gold NPs impacts the degradation. Although Jiang and coworkers found that 40–60 nm NPs resulted in the best performance, no subsequent publication to date has verified this finding or explored the effect of different shapes of NPs. However, different Au nanostructures—such as Au nanorods, stars, or disks—have different internalization properties and likely interact with the cell in different ways.^[^ ^57^, ^58^ ^]^

As Au NPs can easily be functionalized using range of thiols as anchoring ligands, they can be employed to design both monofunctional platforms, as mentioned above, and bifunctional PROTAC platforms. For example, a Au NP system was developed by Wang et al. to harness CRBN to degrade anaplastic lymphoma kinase (ALK), an important target in non‐small cell lung cancer (NSCLC).^[^ ^59^ ^]^ Au nanoclusters (2‐3 nm) were used to degrade HER2 through a PROTAC‐based approach also harnessing CRBN.^[^ ^60^ ^]^ Au nanoclusters have advantages over Au NPs as they are much smaller (2–4 nm) and consequently provide a much higher surface‐to‐volume ratio.

### 3.2 Self‐Assembled Peptide Nanoparticles

Peptides containing specific amino acids, such as diphenylglycine, are known to self‐assemble into nanostructures with diverse morphologies ranging from spheres to nanofibers.^[^ ^61^, ^62^ ^]^ A tetrapeptide containing diphenylglycine, which forms nanospheres (150 nm),^[^ ^61^ ^]^ was recently used to design a multifunctional nanoparticle‐based PROTAC. Yang et al. developed “split‐and‐mix” strategy by combining peptides individually modified with either POI‐binding ligand or the E3‐binding ligand.^[^ ^63^ ^]^ These nanosized peptide assemblies effectively degraded a number of targets including membrane proteins such as EGFR as well as intracellular targets estrogen receptor (ERα), AR, CDK 4/6, MEK1/2, and BCR‐ABL, all of which are critical to cancer progression and metastasis.

The same group further expanded the application of their peptide self‐assemblies by harnessing the autophagy‐lysosomal degradation pathway.^[^ ^42^ ^]^ The “split‐and‐mix” peptide self‐assemblies were modified with the KFERQ peptide sequence that recruits chaperones for autophagy‐mediated degradation.^[^ ^64^, ^65^ ^]^ to degrade a number of different intracellular targets.^[^ ^42^ ^]^

In another study, Wang et al. utilized an amphiphilic peptide‐modified *N*‐acetylgalactosamine (GalNAc) that can self‐assemble into nanospheres and has a strong affinity for asialoglycoprotein receptor targets.^[^ ^66^ ^]^ The nanospheres were modified first with anti‐EGFR antibodies using EDC/NHS coupling, resulting in a nanostructured formulation referred to as Nano‐LYTACs, which were capable of degradation of EGFR. After this preliminary demonstration, the nanospheres were modified with an anti‐CD24 antibodies, and the degradation of CD24 on the surface of macrophages was demonstrated. This CD24 degradation enabled the restoration of a macrophage phagocytosis pathway.

Using a similar technology but different chemistry, Zhang et al. developed in situ self‐assembly approach for peptides that respond to elevated intracellular glutathione levels to form nanoassembled protein degraders. These precursor peptides were functionalized with either a POI‐binding ligand or an E3‐binding ligand, and upon exposure to glutathione, self‐assembled into β‐sheet‐based nanostructures. The resulting supramolecular assemblies spatially arranged the POI and E3 ligands to facilitate targeted protein degradation. These peptide nanoassemblies were successfully employed to degrade a range of membrane protein targets including EGFR and intracellular targets including the androgen receptor (AR).^[^ ^67^ ^]^ Notably, the degradation of EGFR was demonstrated in vivo, highlighting the translational potential of this system, which is currently limited by the need of coadministration of the peptides to ensure effective in situ assembly. Despite this limitation, peptide‐based nanostructures offer a promising direction for future research.

Another in situ self‐assembled PROTAC system was developed by Chen et al. utilizing a sulfatase‐activatable self‐assembly motif to trigger fibril formation.^[^ ^68^ ^]^ The system combined an E3‐binding ligand with a peptide binder of BcL‐xL, a protective antiapoptotic protein. Upon activation by sulfatase, the peptides self‐assembled into fibrils, leading to BcL‐xL degradation, which induced apoptosis and reduced cell viability. The authors further characterized this system in vivo, demonstrating significant reduction of tumor size compared to controls.

In a pioneering approach, Wang et al. introduced LYTACAs (lysosome‐targeting chimeric assemblies), a peptide self‐assembled nanoparticle system that enables the degradation of multiple targets via the lysosomal pathway.^[^ ^69^ ^]^ In their design, mixing of FMOC‐modified IL‐17A‐binding peptides and FMOC scavenger receptor A1 (SR‐A) binding peptides resulted in FMOC‐mediated self‐assembly into spherical nanoparticles (150 nm). Scavenger receptor A1 has been shown to bind and eliminate negatively charged ligands via the lysosome‐dependent pathways.^[^ ^70^ ^]^ Thus, these nanoparticles enhanced the uptake and degradation of the soluble extracellular protein IL‐17A. In an in vivo psoriasis model, this degradation resulted in a reduction in the psoriasis area and severity index (PASI) score and improvements in skin inflammation markers. This design was then extended to an immune check point inhibitor PD‐L1, a common target in cancer immunotherapies. By replacing the FMOC group with Nap‐FF, the authors reduced the size of the nanoparticles to approximately 50 nm, which significantly improved their stability. This nanoparticle system, not only allowed for the efficient degradation of multiple protein targets (soluble and extracellular) but also shifted the focus from oncological targets to inflammatory diseases such as psoriasis.

### 3.3 Liposomes and Membrane Vesicles as Nanoparticle‐Based Degraders

Liposome nanostructures are attractive for biomedical use as they have been employed in number of clinically approved therapeutic strategies and have clear regulatory pathway.^[^ ^44^ ^]^ Following the work on peptide “split‐and‐mix” self‐assemblies, Song et al. designed “split‐and‐mix liposomes”, replacing peptide assemblies with liposomes, and incorporating a cholesterol anchor to ensure outward ligand orientation from the lipid bilayer.^[^ ^51^ ^]^ These LipoSM‐PROTACs were used to degrade an ERα via the E3 VHL.^[^ ^51^ ^]^ In the following year, a proof of concept in vivo study using melanoma mice model demonstrated the efficacy of this liposomal system, showing significant inhibition of tumor growth. The “split‐and‐mix liposomes” were also applied to target MEK1/2 and ALK. Further, in vivo studies confirmed the system's therapeutic potential, demonstrating tumor shrinkage upon MEK1/2 degradation.^[^ ^52^ ^]^

In a different study from Gao et al., they used modified liposomes to leverage the lysosomal degradation system of immune cells for degrading the surface protein SIRPα on macrophages.^[^ ^71^ ^]^ To achieve this, they extruded the red blood cell membranes onto liposomes, enriching the liposome surface with CD47 to enhance targeting. Additionally, the authors combined this system with a Ly6G antibody (aRLP) to increase its accumulation in sites of inflammation. Degradation of SIRPα promoted macrophage efferocytosis of apoptotic cardiomyocytes (CMs) and improved cardiac repair in murine models. This work represents the first nanoparticle degrader to utilize a modified cellular membrane for targeting and degrade a protein to modulate the immune system.^[^ ^71^ ^]^

In a separate study, Wang et al. developed genetically engineered lysosome‐targeting exosomes, referred to as LYTEXs (120 nm).^[^ ^72^ ^]^ These bifunctional exosomes were synthesized with an ASGPR binding motif and either a HER2 or a PD‐L1 binder. The system successfully degraded both proteins and demonstrated in vivo efficacy by degrading PD‐L1 in murine tumor models.

Bacterial outer membrane vesicles were used by Ji et al. to enable PD‐L1 degradation.^[^ ^73^ ^]^ Here, the vesicles (90–110 nm) were engineered to express Lyp1‐traptavidin (a mutant of streptavidin) fusion protein on the surface, thus allowing the attachment of a biotinylated PD‐L1 antibody as well as biotinylated mannose 6‐phosphate ligand. The Lyp1 on the vesicles’ surface enables tumor tissue targeting, enabling degradation of PD‐L1 within the tissue, which results in reduction of tumor size in vivo.

Although modifications can be introduced relatively easily to self‐assembled nanostructures such as liposomes and membrane vesicles by altering individual building blocks, and modular systems can be readily prepared, precise size control remains challenging, and large‐scale manufacturing continues to pose significant hurdles. Despite these limitations, the versatility and potential of self‐assembled nanostructures make them a promising platform for various applications.

### 3.4 Polymeric Nanoparticle‐Based Degraders

Polylactic acid (PLA), polyethylene glycol (PEG), and polystyrene (PS) have all been employed for design of NanoPDs. Among, these, PLA and PEG are FDA‐approved polymers, thus attractive for clinical translation. Polymeric nanostructures offer the flexibility of tailored building blocks, which can be modified with range of functional groups offering a customizable platform for diverse applications.^[^ ^39^, ^74^ ^]^

Yao et al. developed MOdified NanOparticle with TArgeting Binders (MONOTAB) system,^[^ ^48^ ^]^ a polystyrene nanoparticle‐based platform (100 nm) functionalized with streptavidin. Biotin‐functionalized PD‐L1 antibody was then used for NP functionalization, enabling PD‐L1 degradation both in vitro and in vivo and resulting in reduction in tumor volume. The authors further expanded the system by functionalizing the NPs with an anti‐MMP‐2 antibody. As MMP‐2 facilitates cell migration, its degradation resulted in a measurable decrease in cell mobility. Finally, on functionalization with annexin V, which binds phosphatidylserine on the surface of extracellular vesicles, they successfully demonstrated the capture and degradation of extracellular vesicles. This innovative work demonstrates the versatility of the MONOTAB nanoparticle system and its potential for addressing a broad range of extracellular targets, including soluble proteins, membrane‐bound proteins, and vesicles.

In another approach, Liu et al. used polylactic‐co‐glycolic acid (PLGA) nanoparticles (160 nm) for lysosome‐mediated degradation of extracellular proteins.^[^ ^39^ ^]^ By attaching antibody and peptide ligand, they were able to degrade EGFR, PD‐L1, ACE2, and CD‐13.

Huang et al. used 100 nm maleimide–polyethylene glycol–polylactic acid (PEG‐PLA) nanoparticles.^[^ ^53^ ^]^ The nanoparticles were conjugated with a peptide that bound to the target, mutant p53, and a positively charged lipid (DOTAP) to trigger autophagosome formation around the nanoparticle, enabling autophagic degradation. Although further mechanistic analysis is required, in vivo studies demonstrated that the tumor growth inhibition correlated with p53 degradation, highlighting the system's potential.

In a study by Li et al., 50 nm PLGA nanoparticles were functionalized with anti‐PD‐L1 antibodies specific to mice.^[^ ^75^ ^]^ The multivalent binding of the nanoparticles facilitated the internalization and subsequent degradation of PD‐L1, which enhanced the antitumor effects of chemotherapy in vivo.

Utilizing a semiconducting polymer, Xu et al. created nano‐LYTACs to degrade interleukin 4 (IL‐4) receptor in macrophages.^[^ ^76^ ^]^ These nanoparticles were decorated with an IL‐4‐ binding peptide and a lysosomal sorting motif. The core polymer allowed for ultrasound‐induced sonodynamic therapy, which in combination with the immune cell modulation caused by degradation resulted in improved tumor treatment.

A chitosan based “nanosweeper” was designed by Luo et al. Using peptides to bind the target amyloid β‐peptide (Aβ) plaques and an autophagy‐inducing peptide, they were able to degrade amyloid β plaques in mouse models.^[^ ^54^ ^]^

Although polymeric nanosystems, like those described here, offer less control over the particle size compared to inorganic nanomaterials, they provide flexibility in terms of incorporation of functional linkers such as immune‐evasive ligands. However, despite the widespread use of PEG in nanoparticle design, its potential immunogenicity poses challenges.^[^ ^77^ ^]^ Nonetheless, the FDA approval of several polymers underscores their promise as materials for therapeutic applications.^[^ ^44^ ^]^

### 3.5 Self‐Assembled DNA Structures

DNA assembly has emerged as a powerful nanostructuring strategy, particularly with the development of the DNA origami approach. This technique involves folding a long DNA strand into various 2D and 3D shapes with the help of smaller complementary DNA sequences known as staple strands.^[^ ^78^ ^]^ Beyond offering versatility in shape design, DNA origami is highly programmable. By selecting specific sequences and designs, the structures themselves can be precisely controlled by the spacing between ligands attached to the DNA structure. This level of precision is particularly advantageous for designing nanoparticle‐based protein degraders as it minimizes steric hindrance and enhances the efficient binding of ligands to their target proteins. This programmability makes DNA origami an attractive tool for advancing TPD technologies.

The first reported DNA nanostructure used for TPD was a DNA nanotetrahedron designed by Ma et al., which targeted the HER2 receptor using an anti‐HER2 aptamer.^[^ ^37^ ^]^ This 10–15 nm nanostructure successfully degraded HER2 and induced apoptosis in a HER2‐positive cell line. In a later study, Tang et al. modified the DNA tetrahedron structure with cholesterol molecules to facilitate cell membrane binding.^[^ ^79^ ^]^ These nanostructures, measuring 7–13 nm in size, were further modified with an aptamer targeting the tyrosine kinase c‐Met receptor, a protein implicated in cancer signaling and tumor growth.

The precise spatial control offered by DNA origami nanostructures was exploited by Zhou et al. to create DNA framework‐based PROTACs, referred to as DbTACs, to target CDK9 for degradation. Using 10 nm DNA origami, the authors optimized the distance between the CDK9 binder (small molecule) and an CRBN E3 ligand, determining that 26 Å was the ideal spacing for the reaction. This precise positioning likely facilitates the stabilization of the ternary complex. The system was also used to target CDK4/6 for degradation. Remarkably, the DbTACs enabled simultaneous degradation of multiple CDKs (CDK4/6 as well as CDK9) using small molecules, as well as antibodies.^[^ ^41^ ^]^ Additionally, the nanostructures were modified with a DNA‐binding ligand for ETS‐related gene (ERG), a transcription factor oncogene located in the nucleus.

In another system based on DNA nanotetrahedrons, Li et al. created a STAT3 degrader.^[^ ^80^ ^]^ Here, they used a VHL ligand and two DNA based STAT3 binding ligands. They demonstrated degradation in in vivo models and in cell studies.

Utilizing a different DNA design, a circular DNA origami system was introduced by Cui et al. The intelligent modular DNA LYTAC (IMTAC) nanostructure was modified with anti‐EGFR and anti‐PD‐L1 antibodies, both individually, and then in combination.^[^ ^81^ ^]^ The combination demonstrated marked cell death and improved tumor growth inhibition over the monofunctionalized version. This is one of the first dual‐targeted NanoPD for targeted extracellular protein degradation.

### 3.6 Other Nanoparticle‐Based Degraders

There are a few examples of other types of nanostructures used for design of protein degraders. For example, carbon nanoparticles or carbon dots with an average diameter of 3 nm were employed to degrade PD‐L1 using a ligand for the E3 CRBN.^[^ ^82^ ^]^ In in vivo models, PD‐L1 degradation correlated with reduced tumor size and an enhanced immune response.

In another study, Hsu et al. used Nanot nanoparticles, a proprietary nanoparticle system, conjugated to a monoclonal antibody targeting the H1A clone of soluble PD‐L1 (sPD‐L1).^[^ ^49^, ^83^ ^]^ This system effectively bound and depleted sPD‐L1 from the media. Both in vitro and in vivo, the Nanot nanoparticles reduced circulating levels of sPDL‐1 and demonstrated significant tumor size reduction in sPD‐L1 secreting tumors.

A recent LYTAC‐inspired nanoparticle system based on the protein bovine serum albumin achieved degradation of PD‐L1. The ligands are attached via in situ polymerization.^[^ ^84^ ^]^

## 4 Functionalization Strategies in Design of NanoPDs

**Figure 3:**
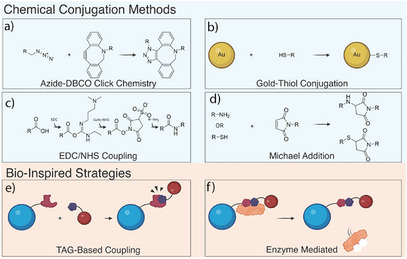
Surface functionalization of NanoPDs. Covalent strategies including a) DBCO‐azide click chemistry, b) gold–thiol conjugation, c) 1‐ethyl‐3‐(3‐dimethylaminopropyl) carbodiimide and *N*‐hydroxysuccinimide (EDC/NHS) coupling, and d) Michael addition with amines or thiol groups. Commonly used bioinspired strategies include e) Tag‐based coupling methods and f) Enzyme‐mediated coupling.

Nanoparticle‐mediated protein degradation platforms rely on the specific attachment of small molecules, peptides, and antibodies to achieve intended functionality. Numerous strategies have been used to conjugate functional moieties to nanoparticle surfaces, and the choice of method will depend both on the ligand of interest and the type of nanoparticles, whereas choice of anchoring groups is limited by nanoparticle type. Choice of the functionalization strategies needs to consider availability of reagents and possible side products, and it needs to be mild, scalable, and reproducible. Both click chemistry and bioinspired conjugation strategies were employed to design ligand‐coated NanoPDs (Figure 3).

### 4.1 Covalent Coupling Strategies

To attach ligands onto nanoparticles, researchers commonly turn to covalent coupling strategies, which result in stable covalent bonds on the nanoparticle surface. The most widely used conjugation strategy in the field of nanoparticle‐mediated targeted protein degradation employs copper‐free click strategy. Here, an azide moiety reacts with a strained alkyne moiety, usually dibenzocyclooctyne (DBCO) group, to form a stable covalent bond.^[^ ^85^ ^]^ Although this reaction is efficient and the reagents readily available, modifying proteins or peptides with either group can be costly. However, many linkers can be either purchased or synthesized.^[^ ^86^ ^]^ The click approach can also employ an azide and unstrained alkyne.^[^ ^86^ ^]^ Although this method is more cost effective, it requires copper catalyst, which requires thorough purification after functionalization due to copper's toxicity to living systems.^[^ ^87^ ^]^ Click chemistry was successfully employed for attachment of peptides, antibodies, and small molecules to a wide range of materials including polymeric, liposomal, and DNA origami nanostructures.

Other types of click reactions, such as trans‐cyclooctene (TCO) and atrazine coupling, could be used as a suitable functionalization strategy due to its fast kinetics and high yields.^[^ ^88^ ^]^ Although the reagents are more expensive and less readily available, the speed and efficiency should not be overlooked.

In the specific case of Au NPs functionalization, thiol‐Au bond formation is often employed as a conjugation strategy. This is particularly useful as many antibodies and peptides used in the design of degraders either contain native cysteine residues or additional cysteines can be engineered into these biomolecules. However, caution is required when multiple cysteines are present as their disruption could lead to unwanted protein unfolding or nonspecific attachment.

Other covalent strategies employed included maleimide–thiol or maleimide–amine reaction,^[^ ^89^ ^]^ as well as EDC/NHS amide coupling, both of which are used to attach small molecules and antibodies to self‐assembled nanostructures and polymer nanoparticles. When quantification of ligands on the surface or the nanoparticle is required, tetrazole‐based photo click reactions that results in fluorescent product could be employed. Although not yet demonstrated in design of NanoPDs, this strategy was successfully used as a self‐reporting conjugation strategy for immobilization of DNA and peptides on the surface of gold nanorods, and it is worth exploring further.^[^ ^90^, ^91^ ^]^

### 4.2 Bioinspired Conjugation Methods

In contrast to covalent coupling strategies, which often rely on harsh chemicals, future conjugation methods will likely need to accommodate the immobilization of sensitive biologics such as antibodies and nanobodies. These biomolecules demand more biocompatible approaches ones that avoid organic solvents or extreme conditions. Such requirements can often be met through bioinspired strategies or through methods that can be genetically encoded to the protein.

In a study employing polystyrene NanoPDs, streptavidin‐labeled nanoparticles and biotin‐labeled anti‐IgG antibodies were used for surface functionalization.^[^ ^48^ ^]^ Streptavidin–biotin strategy, widely used in design of bio–nano hybrids, allows for strong attachment of biologics in a modular way. For example, another study utilized genetically encoded bacterial outer membrane vesicles to express traptavidin, a streptavidin mutant, allowing for biotin‐functionalized binders to be attached in a modular fashion.^[^ ^73^ ^]^

Although not yet employed in NanoPD field, protein tag strategies could be useful as they can be genetically encoded and adapted to different classes of proteins. Useful tag approach is the SpyCatcher system, where a small peptide (SpyTag) binds to a SpyCatcher protein derived from the CnaB2 domains of the FbaB protein from *Streptococcus pyogenes* and forms an ultrahigh affinity bond. This approach has been used previously to enable precise positioning of biomolecules on the nanoparticle surface^[^ ^92^, ^93^ ^]^ and is particularly advantageous due to the ease by which the SpyCatcher can be genetically encoded within protein ligands.

Similarly, other tag systems have been utilized in the past to modify nanoparticles and could be adapted to the NanoPD design. For example, the SNAP Tag system, based on covalent bond formation between a benzyl‐guanine group and modified form of the DNA repair enzyme human O6‐alkylguanine‐DNA‐alkyltransferase,^[^ ^94^ ^]^ has been used to attach proteins to various nanoparticles.^[^ ^95^ ^]^

The HALO tag system, where a halogenated linker reacts with a dehalogenase enzyme,^[^ ^96^ ^]^ has been used to functionalize range of nanoparticles in a site‐specific way.^[^ ^97^ ^]^ Other tag systems such as polyHistidine (His)^[^ ^98^ ^]^ and streptadivin (STEP) tags,^[^ ^99^ ^]^ widely employed in purification of proteins, might be useful strategies when other tag strategy impact the integrity of the protein binders.

Two other bioinspired strategies that might be employed for functionalization of nanoparticle degraders include split inteins (already used to conjugate quantum dots in living systems^[^ ^100^ ^]^) and DNA‐directed immobilization.^[^ ^101^, ^102^ ^]^ Although the split intein strategy utilizes polypeptide sequences capable of self‐excision and stable bond formation, DNA‐directed immobilization relies on DNA hybridization, requiring complementary DNA strands to be attached to both the nanoparticles and the target biomolecule.

Finally, given the need for specific and mild attachment of biologics, valuable insights can be drawn from the field of antibody–drug conjugates (ADC). One promising approach is enzyme‐guided coupling, which enables the replacement of the antibody's natural sugar with a modified sugar, offering significant potential for design of antibody‐modified NanoPDs.^[^ ^103^ ^]^ Another involves tyrosine binders, which utilize specific reactive groups such as 4‐phenyl‐3*H*‐1,2,4‐triazoline‐3,5(4*H*)‐diones (PTADs), selectively targeting tyrosines.^[^ ^104^ ^]^

Given the successes in precise control over nanoparticle surface modification, other aspects of ligand decoration, such as loading density or the distance between bifunctional moieties and the nanoparticle's surface, could also be fine‐tuned. This is particularly relevant as the protein corona (Section 5) may affect the ubiquitination reaction, potentially leading to the unintended ubiquitination of nonspecific corona proteins instead of the target. It would be interesting to explore whether PEGylation disrupts or enhances this process and whether strategies that position proteins further from the nanoparticle surface help minimize nonspecific ubiquitination. Additionally, as ternary complex formation is a well‐established key aspect of the PROTAC mechanism, it is crucial to investigate how the NanoPD system influences this process, which is an often overlooked phenomenon.^[^ ^41^ ^]^

## 5 Challenges and Opportunities of Nanoparticle‐Mediated Targeted Protein Degradation

**Figure 4:**
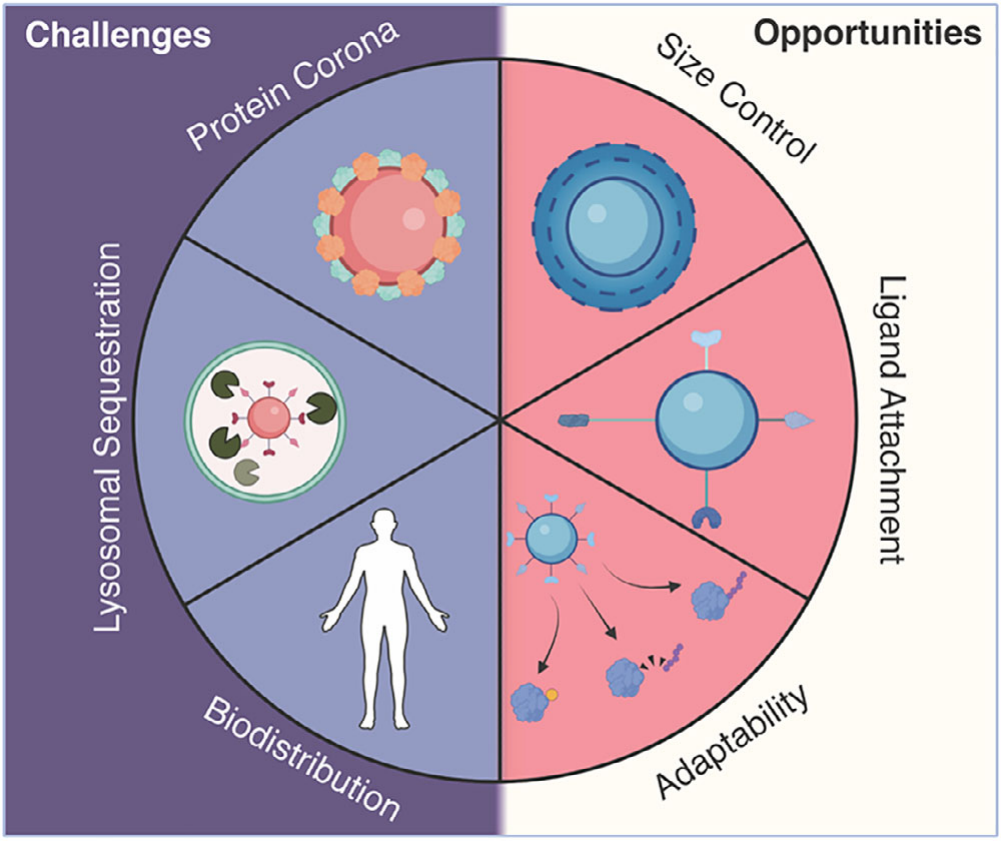
Challenges associated with nanomaterials in medicine include biodistribution, protein corona formation, and lysosomal sequestration, whereas key advantages include precise size control, ligand attachment, and adaptability.

Although nanoparticle‐mediated targeted protein degradation holds great potential to transform the field, it also faces challenges inherent to nanoparticles in general. These include lysosomal sequestration, protein corona formation, proper orientation of ligands during conjugation, and unknown degradation pathways in vivo (Figure 4). Although several nanoparticle systems have been clinically approved,^[^ ^44^ ^]^ mostly for enhancing delivery of hydrophobic drugs, prolonging circulation times, or improving imaging contrast, developing efficient and reliable targeting strategies remains a significant challenge.

### 5.1 Biodistribution and Tumor Targeting

Various biological barriers hinder the nanoparticle journey from the injection site to the tumor.^[^ ^105^ ^]^ Although nanoparticles can enhance the circulation time and stability of small molecules due to their size and shape, they are also prone to clearance by liver sinusoidal endothelial cells (LSECs) and macrophages, which direct them into the reticuloendothelial system (RES).^[^ ^105^, ^106^ ^]^Consequently, nanoparticles frequently accumulate in the liver and are rapidly cleared from circulation. If they reach the kidneys, the extensive fenestration and porous network can lead to either entrapment or rapid elimination for nanoparticles smaller than 8 nm, further limiting their delivery efficiency.^[^ ^105^ ^]^

Additional barriers further impede nanoparticle uptake into tumors, which are common targets for nanocarrier systems. In the early days of nanomedicine, it was hypothesized that nanoparticles and proteins passively accumulate in tumors due to the presence of leaky blood vessels, a phenomenon known as the enhanced permeability and retention (EPR) effect.^[^ ^107^ ^]^ Although widely accepted, this hypothesis was recently challenged,^[^ ^108^ ^]^ prompting researchers to explore active uptake mechanisms, collectively referred to as the active transport and retention principle.^[^ ^109^, ^110^, ^111^ ^]^

Moreover, it became clear that nanoparticle dosing plays a crucial role in tumor accumulation. Studies suggest that approximately one trillion nanoparticles are required to saturate the RES system and achieve effective tumor targeting.^[^ ^112^ ^]^ However, many studies overlook this threshold as the molar concentration of nanomedicines is rarely reported during the design of tumor‐targeting strategies. This gap highlights the need for more precise quantification and optimization in nanoparticle‐based therapeutic approaches.

Despite the in vitro successes of targeting nanoparticles, their efficacy in enhancing tumor accumulation in vivo remains uncertain.^[^ ^113^ ^]^ Emerging studies suggest that nanoparticles modified with targeting molecules do not significantly improve tumor penetration compared to their unmodified counterparts.^[^ ^114^ ^]^ Furthermore, the absence of clinically approved targeted nanoparticles^[^ ^44^ ^]^ underscores the need to rethink current strategies and carefully weigh the cost‐benefit trade‐offs of extensive nanoparticle modifications.^[^ ^115^ ^]^

This lack of conclusive evidence regarding the effectiveness of targeting strategies should be a key consideration in the design of nanoparticle‐mediated protein degraders, particularly for solid tumors, where many existing approaches have proven ineffective. Researchers in this field must integrate insights from other areas of nanomedicine, prioritizing critical factors such as dosing and material selection. Emphasis should be placed on systems already in clinical trials or with regulatory approval to accelerate the translation of these technologies into practical applications.

### 5.2 Protein Corona and Nanoparticle Stability

When nanoparticles are introduced into a protein solution, a protein corona can form on their surface due to surface charge or activity.^[^ ^116^, ^117^ ^]^ This phenomenon significantly impacts nanoparticle cellular uptake, immune sequestration, and clearance.^[^ ^105^ ^]^ Although protein corona formation has been extensively explored, demonstrating both beneficial and detrimental effects,^[^ ^118^ ^]^ its impact on nanoparticle‐mediated protein degradation remains largely unexplored. Protein corona formation could profoundly influence the degradation of target proteins and the overall efficiency of such systems, particularly those relying on precise protein binding and interactions with molecules on the nanoparticle surface. Strategies to mitigate protein corona formation, such as pretreating nanoparticles with inert proteins like albumin^[^ ^118^ ^]^ or incorporating polyethylene glycol (PEG) on the surface, have been investigated. However, these approaches primarily reduce rather than eliminate corona formation, highlighting the need for further research into its implications for nanoparticle‐mediated protein degradation.

### 5.3 Endosomal Sequestration

One of the major challenges in using nanocarriers is lysosomal sequestration, in particular when delivering proteins or nucleic acids that need to reach the cytosol and nucleus.^[^ ^119^, ^120^ ^]^ Studies estimate that only 1%–2% of administered nanoparticles successfully escape into the cytosol.^[^ ^121^ ^]^ Lysosomal escape remains an active area of research, with strategies such a proton sponges and fusogenic lipids showing promise.^[^ ^122^, ^123^ ^]^ However, these approaches have not been widely adopted and further studies are needed to optimize their effectiveness and broaden their applicability.

Lysosomal sequestration affects nearly all nanoparticles, regardless of type, size, or morphology, and likely affects the degradation of cytosolic proteins. Although extensive studies have demonstrated the degradation of surface proteins through lysosomal pathway, it remains unclear how similar nanomaterials can effectively degrade intracellular proteins, given the low lysosomal escape rate of 1%–2%.^[^ ^124^ ^]^

To advance the field of protein degradation, more research is needed to better understand degradation pathways. Nanoparticle‐mediated protein degradation would particularly benefit from high‐resolution imaging techniques to study and quantify nanoparticle escape, protein binding within the cell, and the intracellular formation of the ternary complex.

Despite these challenges, nanoparticle‐mediated protein degradation offers significant opportunities. The size and shape of many nanostructures can be precisely controlled, surface chemistry can be fine‐tuned, and the intrinsic properties of nanomaterials can be harnessed to enhance degradation. For example, light‐activated or magnetic‐field‐induced hyperthermia could be leveraged to promote protein degradation or facilitate lysosomal escape, paving the way for therapeutic innovation.

### 5.4 Impact of Nanoparticle Size and Shape

Over the past decades, significant progress has been made to control the size and shape of nanoparticles, with protocols enabling the reproduceable and scalable production of various nanomaterials. Although achieving tunable nanoparticles remains challenging for organic systems such as liposomes or protein‐based nanoparticles, significant advances have been made with polymeric and inorganic systems. To date, Jiang et al. have conducted the only study exploring the role of Au NPs size in particle internalization and protein degradation, demonstrating that the optimal effect is achieved with 40–60 nm particles, as determined by TEM.^[^ ^36^ ^]^ Similar studies should be extended to other types of nanoparticles as extensive studies have shown that nanoparticles size, stiffness, and other physical properties define the endocytosis pathway taken and cellular interactions.^[^ ^125^ ^]^

Given that size, shape, and material stiffness strongly influence nanoparticle uptake mechanisms,^[^ ^125^ ^]^ we anticipate that alternative endocytosis pathways, such as micropinocytosis and other engulfment pathways, could be utilized to facilitate degradation strategies. This is particularly relevant for strategies like MADTACs, where proteins are degraded through the macroautophagy pathway.^[^ ^35^ ^]^ Notably, these cellular interactions and degradation mechanisms could be modulated simply by adjusting nanoparticles size and shape, presenting new opportunities for targeted protein degradation.

## 6 Looking to the Future: Lessons Learned and Next Steps

### 6.1 Targeted Nanoparticles as Mediators of Antigen Depletion

The field of nanoparticle‐mediated protein degradation has repeatedly demonstrated that monofunctionalized nanoparticles, designed for target binding, can induce target degradation. This well‐established finding highlights the significant potential of targeted extracellular protein degradation and has been successfully replicated by numerous research groups.

However, it also raises important questions about the existing paradigm of nanoparticle targeting strategies as monofunctionalized nanoparticles have long been employed in targeted delivery. Given current state‐of‐the‐art, it appears that simple monofunctionalized nanoparticles may inadvertently deplete the targeted antigens on the cells surface, much like that in antibody drug conjugates or CART cell therapies, where antigen depletion causes resistance to these therapies.^[^ ^126^, ^127^ ^]^ Therefore, further research is essential to better characterize and understand this phenomenon, particularly in the context of targeted delivery of nanotherapeutics and contrast agents in the clinic.

### 6.2 Biocompatibility and Biodistribution

Thanks to progress in the nanocarrier field and the clinical of several classes of nanoparticles, our understanding of nanoparticle biocompatibility and biodistribution has significantly improved, facilitating more informed choices in selecting NanoPD materials. However, several critical unknowns remain, including the impact of protein corona formation, the role of the linker length and density, and the ultimate fate of nanoparticles postadministration.^[^ ^105^ ^]^ Addressing these challenges will be crucial for optimizing nanoparticle‐based protein degradation strategies and advancing their clinical translation.

### 6.3 Mechanism of Nanoparticle‐Mediated Targeted Protein Degradation

The exact mechanism by which the nanomaterial itself affects nanoparticle‐mediated protein degradation remains unclear. One recent study suggested that the large surface area of nanoparticles enables multiple binding events, allowing them to function similarly to bifunctional molecules or multivalent polymers.^[^ ^36^ ^]^ Previous nanoparticle‐uptake studies have employed CRISPR‐based knockout strategies to identify key surface proteins involved in gold nanoparticle internalization. These studies identified ApoB–LDL receptor and complement C8‐LDL as primary mediators of uptake.^[^ ^128^ ^]^ Another study used a genome‐wide forward genetic screening to show that the scavenger receptor SCARB1 and the proteoglycan surface marker heparan sulfate play critical roles in silica nanoparticle uptake.^[^ ^129^ ^]^ Although some of these receptors, such as LDL and SCARB1, have been explored in the context of nanoparticle‐mediated targeted protein degradation,^[^ ^48^ ^]^ numerous other surface proteins implicated in nanoparticle uptake remain underexplored. Further research into these newly identified mediators could provide valuable insights for optimizing nanoparticle‐based degradation strategies.

In the nanoparticle‐mediated targeted protein degradation field, recent study found that none of the tested receptors, including insulin‐like growth factor 2 receptor (IGF2R), asialoglycoprotein receptor (ASGPR), and scavenger receptor (SCARB1), independently facilitated nanoparticle uptake.^[^ ^48^ ^]^ Moreover, even after protease‐mediated degradation of surface receptor proteins, only a slight reduction in nanoparticle uptake was observed, suggesting that uptake is primarily a physical process governed by nanoparticle cell interactions rather than receptor‐mediated endocytosis.^[^ ^48^ ^]^

Future studies should focus on elucidating the physical interactions that drive nanoparticle uptake and protein degradation. Key factors such as nanoparticle size, surface charge, and material composition must be systematically explored to refine our understanding of nano–bio interactions and optimizing nanoparticle‐mediated protein degradation strategies.

### 6.4 Binding Epitopes and Multivalency

A recent study on extracellular protein degradation using antibody‐PROTACs (AbTACs) has shown that the epitope to which an antibody binds is the most critical factor for effective extracellular target degradation.^[^ ^31^ ^]^ Interestingly, specific binding epitopes were found to be more critical than the overall binding affinity of antibodies for their target proteins.^[^ ^5^, ^31^ ^]^ For example, antibodies binding closer to the cell membrane, at the stalk region of a receptor, were ten times more effective at inducing degradation that those binding to the head domain.^[^ ^5^, ^31^ ^]^ A similar trend was observed with KineTACs.^[^ ^5^, ^24^ ^]^ Given these findings, nanoparticle‐based strategies should explore targeting different antibody epitopes, simultaneously engaging multiple epitopes or leveraging multivalency to enhance degradation efficiency.

Multivalency itself may drive increased endocytosis and degradation. Some studies on multivalent polymers have demonstrated that higher valency enhances endocytosis rates compared to single antibodies.^[^ ^130^ ^]^ A similar effect could occur in nanoparticle systems, where multiple binding sites increase affinity through avidity. As a result, nanoparticle‐mediated protein degradation of extracellular proteins may be influenced by avidity effects, the simultaneous binding of multiple target molecules, or direct physical interactions between the nanoparticle and membrane proteins, potentially triggering degradation or a combination of these mechanisms.^[^ ^130^ ^]^

### 6.5 Nanoparticles and PROTAC Ternary Complex Formation

For intracellular protein degradation, current PROTACs face dose‐limiting challenges, known as the “hook effect”, where high PROTAC concentrations reduce degradation efficiency.^[^ ^131^ ^]^ This occurs when excess PROTACs favor the formation of PROTAC‐POI/E3 binary complex instead of E3 ligase‐PROTAC‐POI ternary complexes required for ubiquitination. Controlling the concentration and spatial distribution of the two types of ligands molecules on nanoparticles surfaces could help mitigate this challenge.

Additionally, the distance between the POI and the E3 ligase is crucial. A DNA nanoparticle study found that an optimal spacing of 26 Å resulted in the most effective in vitro degradation.^[^ ^41^ ^]^ However, further studies are needed to determine whether this finding is specific to the particular POI‐E3 pair studied or if it represent a broader principle. Future research should also explore linker strategies, examining properties such as stiffness or length, as these factors could significantly influence degradation outcomes.

PROTACs present medicinal chemistry challenges as each POI requires a unique ligand and a tailored linker design to connect it to the degradation‐targeting ligand. In contrast, nanoparticle‐mediated protein degradation offers a more flexible and scalable approach. It requires only a single anchoring linker with a functional group that can be easily modified to attach degrader ligands, for instance, via click chemistry. This modular design allows for the rapid synthesis and screening of multiple nanoparticle systems. With over 600 E3s potentially available for use with nanoparticle degraders,^[^ ^13^, ^14^, ^132^ ^]^ the demand for screening is substantial, but so is the opportunity to identify more effective targets and optimized degrader systems. Additionally, although many E3 ligases currently lack small‐molecule ligands, they do possess natural peptide ligands (short “degron” sequences from their native substrates). These peptide ligands could be easily functionalized and attached to the nanoparticles surface for high‐throughput screening, further accelerating the discovery of novel degradation strategies.^[^ ^14^ ^]^

### 6.6 Nanoparticle Mediators of Proximity‐Induced Biology

TPD is one class of proximity‐induced biology, but the potential of nanoparticles extends well beyond this class of reaction. Nanoparticles could serve as versatile scaffolds to mediate a wide array of proximity‐driven post‐translational modifications, such as phosphorylation,^[^ ^133^ ^]^ dephosphorylation,^[^ ^134^ ^]^ nonproteolytic ubiquitination,^[^ ^135^ ^]^ deubiquitination,^[^ ^136^ ^]^ O‐GlcNAcylation (O‐linked *N*‐acetylglucosamine),^[^ ^137^ ^]^ and acetylation of POIs.^[^ ^138^, ^139^, ^140^, ^141^, ^142^ ^]^ Many of the strategies identified here could be adapted for this purpose, enabling the precise modulation of various biological processes.

An example of this is the nanoparticle system developed by Duan et al., which effectively desensitized mast cells to allergen‐induced degranulation.^[^ ^143^ ^]^ In this approach, the nanoparticles inhibited the typical mast cell immune response by simultaneously engaging IgE receptors (using trinitrophenol TNP) and binding to the inhibitory protein siglec‐8. Rather than inducing protein degradation, the mechanism relied on nanoparticle‐mediated proximity, which facilitated the interaction between IgE and siglec‐8 receptors on immune cells surface.^[^ ^25^ ^]^ This study, and others like it, highlights the broader potential of nanoparticles to orchestrate protein–protein interactions, opening new avenues for modulating cellular behavior through proximity‐induced biology.^[^ ^25^, ^143^, ^144^ ^]^

### 6.7 Future of Nanoparticle‐Mediated Targeted Protein Degradation

Currently, most nanoparticle‐mediated protein degradation strategies are predominantly focused on oncology. However, this scope is expected to expand into areas such as immunology and diseases where protein degradation may prove beneficial, including inflammatory disorders, neurological diseases, and antiviral therapies, mirroring the advancements seen with small molecule PROTACs.^[^ ^6^ ^]^ Furthermore, nanoparticles may outperform small molecules in specific contexts, such as preferential accumulation in lysosomes or macrophages at the cellular level or targeted delivery to organs like the liver, kidney, or spleen.

The field of nanoparticle‐mediated protein degradation is rapidly advancing and holds immense potential. However, it faces many of the same challenges that have long constrained the field of nanomedicine. By drawing on existing knowledge and addressing these hurdles, this emerging field presents significant opportunities to improve our understanding of protein degradation and expand applications in proximity‐induced biology. By leveraging the strengths of both nanotechnology and targeted protein degradation, researchers can develop innovative nanoparticle‐based therapeutics with the potential to improve disease outcomes across a wide range of diseases.

## Conflict of Interests

The authors declare no conflict of interest.

## Data Availability

Data sharing is not applicable to this article as no new data were created or analyzed in this study.
